# Alteration of EGFR Spatiotemporal Dynamics Suppresses Signal Transduction

**DOI:** 10.1371/journal.pone.0039682

**Published:** 2012-06-27

**Authors:** Harmony F. Turk, Rola Barhoumi, Robert S. Chapkin

**Affiliations:** 1 Program in Integrative Nutrition and Complex Diseases, Texas A & M University, College Station, Texas, United States of America; 2 Image Analysis Laboratory, Texas A & M University, College Station, Texas, United States of America; 3 Center for Environmental and Rural Health, Texas A & M University, College Station, Texas, United States of America; Cornell University, United States of America

## Abstract

The epidermal growth factor receptor (EGFR), which regulates cell growth and survival, is integral to colon tumorigenesis. Lipid rafts play a role in regulating EGFR signaling, and docosahexaenoic acid (DHA) is known to perturb membrane domain organization through changes in lipid rafts. Therefore, we investigated the mechanistic link between EGFR function and DHA. Membrane incorporation of DHA into immortalized colonocytes altered the lateral organization of EGFR. DHA additionally increased EGFR phosphorylation but paradoxically suppressed downstream signaling. Assessment of the EGFR-Ras-ERK1/2 signaling cascade identified Ras GTP binding as the locus of the DHA-induced disruption of signal transduction. DHA also antagonized EGFR signaling capacity by increasing receptor internalization and degradation. DHA suppressed cell proliferation in an EGFR-dependent manner, but cell proliferation could be partially rescued by expression of constitutively active Ras. Feeding chronically-inflamed, carcinogen-injected C57BL/6 mice a fish oil containing diet enriched in DHA recapitulated the effects on the EGFR signaling axis observed in cell culture and additionally suppressed tumor formation. We conclude that DHA-induced alteration in both the lateral and subcellular localization of EGFR culminates in the suppression of EGFR downstream signal transduction, which has implications for the molecular basis of colon cancer prevention by DHA.

## Introduction

The epidermal growth factor receptor (EGFR;ErbB1) is a transmembrane receptor tyrosine kinase, which contains an extracellular binding domain, a single transmembrane spanning domain, and a cytoplasmic tyrosine kinase domain [1], [2]. Ligands for EGFR, including EGF, bind to the extracellular domain of EGFR, stimulating conformational changes that support receptor dimerization. Receptor dimerization results in the activation of the intracellular tyrosine kinase domain, which phosphorylates the dimerization partner on specific tyrosine residues. The phosphorylated tyrosine residues then function as docking sites for adaptor proteins, which serve to activate intracellular signaling cascades. Ultimately, these cascades result in alterations of gene expression, which determines the biological response to receptor activation.

Key to the ability of EGFR to activate downstream pathways is its localization in lipid raft domains of the plasma membrane [3], [4], [5], [6], [7]. Lipid rafts are highly-ordered, detergent-resistant membrane domains enriched in cholesterol, sphingolipids, and saturated fatty acyl chains that function as signaling platforms [8]. Localization of EGFR to lipid rafts is crucial for efficient EGFR signaling, partially due to colocalization with downstream mediators within lipid rafts [9], [10]. In addition, disruption of lipid rafts results in the relocalization of EGFR to bulk membrane regions, which alters EGFR activation and signaling [5], [6], [9], [11], [12], [13]. Therefore, it is likely that these specialized membrane domains provide a mechanism for spatial and temporal control of EGFR signaling.

Aberrant expression or activation of EGFR has been strongly linked to the etiology of several human epithelial cancers, including colon cancer [14]. Colon cancer is a major public health concern, being the third leading cause of cancer related deaths in the United States [15]. Signaling through EGFR activates various cellular processes involved in carcinogenesis, such as cell proliferation, inhibition of apoptosis, angiogenesis, cell motility, and metastasis [16], [17]. The numerous signaling cascades that radiate from EGFR, including the Akt, extracellular signal regulated kinase (ERK) 1/2, and signal transducer and activator of transcription (STAT) 3 pathways, mediate a variety of mitogenic, metastatic, and other tumor-promoting cellular activities. Signaling through EGFR is up-regulated in colon cancer [18], and inhibition of signaling through EGFR has been shown to prevent colon tumor formation [19]. Additionally, overexpression of EGFR has been reported in up to 85% of human colon cancers [20], [21], [22], [23], [24], and expression of EGFR in colon cancer is correlated with a more aggressive disease and poor patient prognosis [25], [26], [27], [28]. Collectively, these data implicate EGFR as a master signal capable of driving colon tumorigenesis. For these reasons, EGFR is an attractive target for therapeutic intervention; thus, intense efforts have been made to inhibit the activity of EGFR by designing small molecules against the tyrosine kinase domain (erlotinib, gefitinib, and lapatinib) or antibodies against the ligand binding domains (cetuximab and panitumumab) [29], [30].

Importantly, there is substantial experimental, epidemiological, and clinical evidence suggesting that consumption of n-3 polyunsaturated fatty acids (PUFA), including docosahexaenoic acid (DHA, 22∶6^Δ4,7,10,13,16,19^) and eicosapentaenoic acid (EPA, 20∶5^Δ5,8,11,14,17^) is protective against colon tumorigenesis [31], [32], [33], [34], [35], [36], [37], [38], [39], [40], [41], [42], [43], [44], [45]. However, the exact mechanisms by which n-3 PUFA function as chemopreventive agents have not been fully elucidated. Recent evidence suggests that perturbation of cell signaling events emanating from lipid rafts could be one mechanism of action of n-3 PUFA, specifically DHA [46], [47], [48]. DHA can profoundly influence cellular membrane composition and has been shown to have significant effects on plasma membrane properties, including membrane fluidity, phase behavior, permeability, fusion, flip-flop, and protein function following incorporation into membrane phospholipids [48], [49]. Due to its high level of unsaturation, DHA has very poor affinity for cholesterol, which is enriched in lipid raft regions of the plasma membrane [50]. Studies conducted in various cell types have shown that treatment with DHA can alter the size of lipid rafts as well as signaling that is known to occur within rafts [47], [51], [52], [53]. Furthermore, evidence suggests that treatment of cells with DHA results in exclusion of certain proteins from lipid rafts causing disruption of signal transduction events [46]. Therefore, we hypothesized that DHA exerts its effect on colon cancer in part by altering the localization and signaling of EGFR.

Two initial studies have assessed the effects of n-3 PUFA on EGFR localization and signaling. Initially, a combination of EPA and DHA was shown to alter the localization of EGFR within the plasma membrane of a breast cancer cell line. The altered localization was concurrent with increased ligand-induced EGFR phosphorylation and downstream activation of p38MAPK [54]. The second study utilized DHA alone, and similarly reported an alteration in EGFR localization in lung and breast cancer cell lines [55]. Although EGFR phosphorylation and downstream signaling was only investigated under basal conditions, the increase in EGFR phosphorylation was associated with a suppression of Ras and ERK1/2 activation upon DHA treatment. This observation, although intriguing, did not directly address how n-3 PUFA impact EGFR functionality since receptor activation was never examined within the context of a ligand specific for the EGFR. Therefore, it is ambiguous whether the suppressive effects observed are due to alterations in EGFR or other parallel signaling pathways. Additionally, these studies only assessed a single downstream cascade from EGFR. Consequently, we set out to clarify the precise effect of n-3 PUFA on EGFR signaling in the colon.

In this study, we demonstrate that treatment with DHA alters the localization of EGFR within the plasma membrane. Additionally, DHA induces an increase in ligand-induced EGFR phosphorylation with a paradoxical decrease in downstream signaling. These results reveal a novel mechanism by which DHA suppresses cell signaling by perturbing localization of key signaling mediators within lipid rafts. The suppressive effect on EGFR signaling is enhanced by DHA-induced increases in EGFR internalization and degradation. Furthermore, our data demonstrate an uncoupling of receptor phosphorylation from downstream signaling that may affect our understanding of membrane localized receptor-mediated signaling events.

## Results

### DHA Reduces Partitioning of EGFR into Lipid Raft Domains

**Figure 1:**
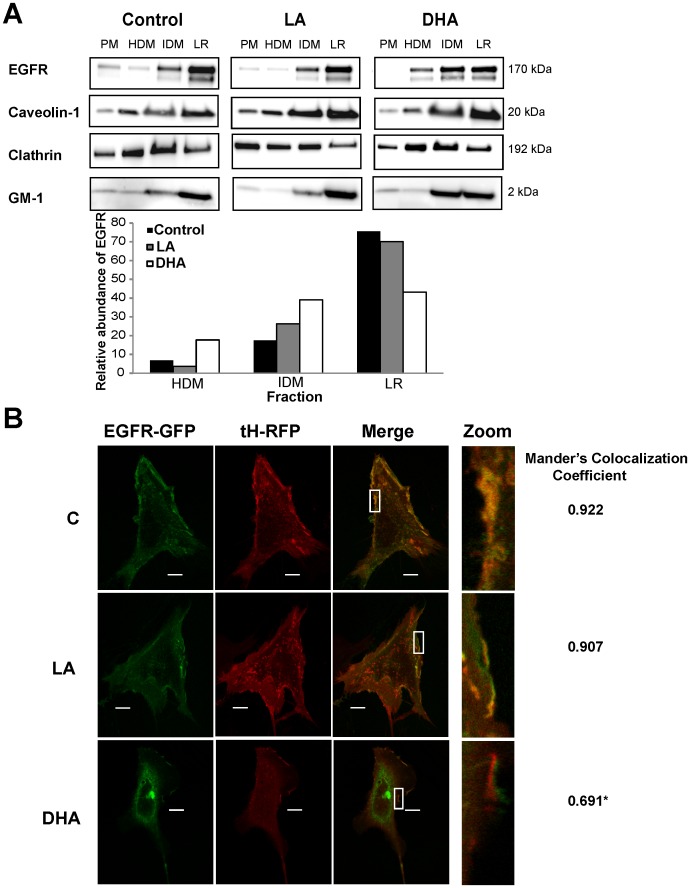
DHA reduces localization of EGFR to lipid rafts. A) YAMC cells were untreated (control) or treated with 50 µM BSA-complexed fatty acids (LA or DHA) for 72 h (12 flasks per treatment). For the final 16–18 h, cells were incubated with low serum media (0.5% FBS) with the same concentration of fatty acids. Cells were harvested from each flask, pooled (n = 12), and the plasma membrane (PM) was isolated. Following isolation, the plasma membrane was fractionated into 3 distinct fractions, high density membrane (HDM), intermediate density membrane (IDM), and lipid raft enriched membrane (LR) by gradient ultracentrifugation. Fractions were collected and an equal amount of protein from each fraction was analyzed by Western blotting using antibodies against EGFR, caveolin-1, and clathrin or using peroxidase conjugated cholera toxin B subunit (for GM-1). Quantification of band intensity was performed, and data are presented as the relative amount of EGFR in each fraction, with the sum of each fraction equaling 100. Western blots are representative of 2 independent experiments. C, control; LA, linoleic acid; DHA, docosahexaenoic acid; PM, plasma membrane; HDM, high density membrane; IDM, intermediate density membrane; LR, lipid raft enriched membrane. B) YAMC cells were treated with 50 µM BSA-complexed fatty acids for 72 h. Twenty-four h after initiating fatty acid treatment, cells were co-transfected with RFP-tH and EGFR-mGFP. Approximately 32 h after transfection, cells were incubated in low serum media (0.5% FBS) overnight prior to imaging. Images are representative of 4 independent experiments. Whole cell images of each individual channel and the merged images are shown on the left. High magnification images of the plasma membrane are shown on the right. Mander’s colocalization coefficient was calculated at the plasma membrane for the amount of EGFR-mGFP (green) colocalizing with RFP-tH (red) using Nikon Elements AR 3.2. The coefficient is the mean of n = 30–40 cells per treatment. Statistical significance between treatments (*P<0.05) was determined using ANOVA and Tukey’s test of contrast. Bars,10 µm.

Lateral organization of EGFR within the plasma membrane is directly linked to receptor function. Previous reports have shown that n-3 PUFA can alter the localization of EGFR within the plasma membrane of lung and breast cancer cells [54], [55]. Therefore, we first determined the effect of DHA on localization of EGFR within the plasma membrane of colonocytes. Initial experiments were conducted using the immortalized Young Adult Mouse Colonocyte (YAMC) cell line, originally described by Whitehead and colleagues [56]. We employed two complementary, established methods to investigate the localization of EGFR. First, a detergent-free method of plasma membrane fractionation to isolate lipid raft enriched domains was utilized [57]. We chose to use a detergent-free method due to strong evidence suggesting that detergent-based methods of extraction can lead to artifacts [58]. Cells were either untreated (control; no fatty acid) or incubated with 50 µM DHA or linoleic acid (LA, 18∶2n−6; control fatty acid) for 72 h. For the final 16–18 h of fatty acid treatment, cells were incubated in low serum media (0.5% FBS) prior to harvesting because EGFR has been shown to be localized to lipid rafts prior to activation. The bulk plasma membrane was isolated and fractionated on density gradients into three fractions, and each fraction was analyzed using Western blotting. The lowest density fraction from each treatment was enriched with caveolin and GM-1, which served as lipid raft markers, and depleted of clathrin, which is excluded from lipid rafts, indicating that this fraction is enriched with lipid rafts. In control and LA treated cells, the majority of EGFR was concentrated in the lipid raft enriched fraction of the plasma membrane (Figure 1A). However, in DHA treated cells, EGFR displayed a more even distribution across all plasma membrane fractions, indicating a lateral reorganization of EGFR. Additionally, confocal fluorescence microscopy was used to determine localization of EGFR. Control and fatty acid treated cells were co-transfected with monomeric GFP-tagged EGFR (EGFR-mGFP) and RFP-tagged truncated H-Ras (RFP-tH). RFP-tH, which is targeted by a CAAX motif that is both palmitoylated and farnesylated, is a well-established lipid raft marker that is localized exclusively to low density fractions on sucrose gradients [59], [60]. In control and LA treated cells, plasma membrane EGFR-mGFP strongly colocalized with RFP-tH (Figure 1B). This colocalization was significantly decreased by treatment with DHA. Interestingly, in many of the DHA treated cells, EGFR-mGFP and RFP-tH formed patches that were localized in close proximity, but not overlapping. The overall localization of the lipid raft marker, RFP-tH, was altered by treatment with DHA, which is in agreement with a plethora of data suggesting that DHA alters lipid rafts [51]–[53]. Additionally, a large portion of EGFR in the DHA-treated cells was localized intracellularly. These complementary datasets indicate that DHA treatment altered the plasma membrane organization of EGFR within colonocytes, which is consistent with previous observations in breast and lung cells [54], [55].

### DHA Suppresses EGFR Signaling but Increases EGFR Phosphorylation

**Figure 2:**
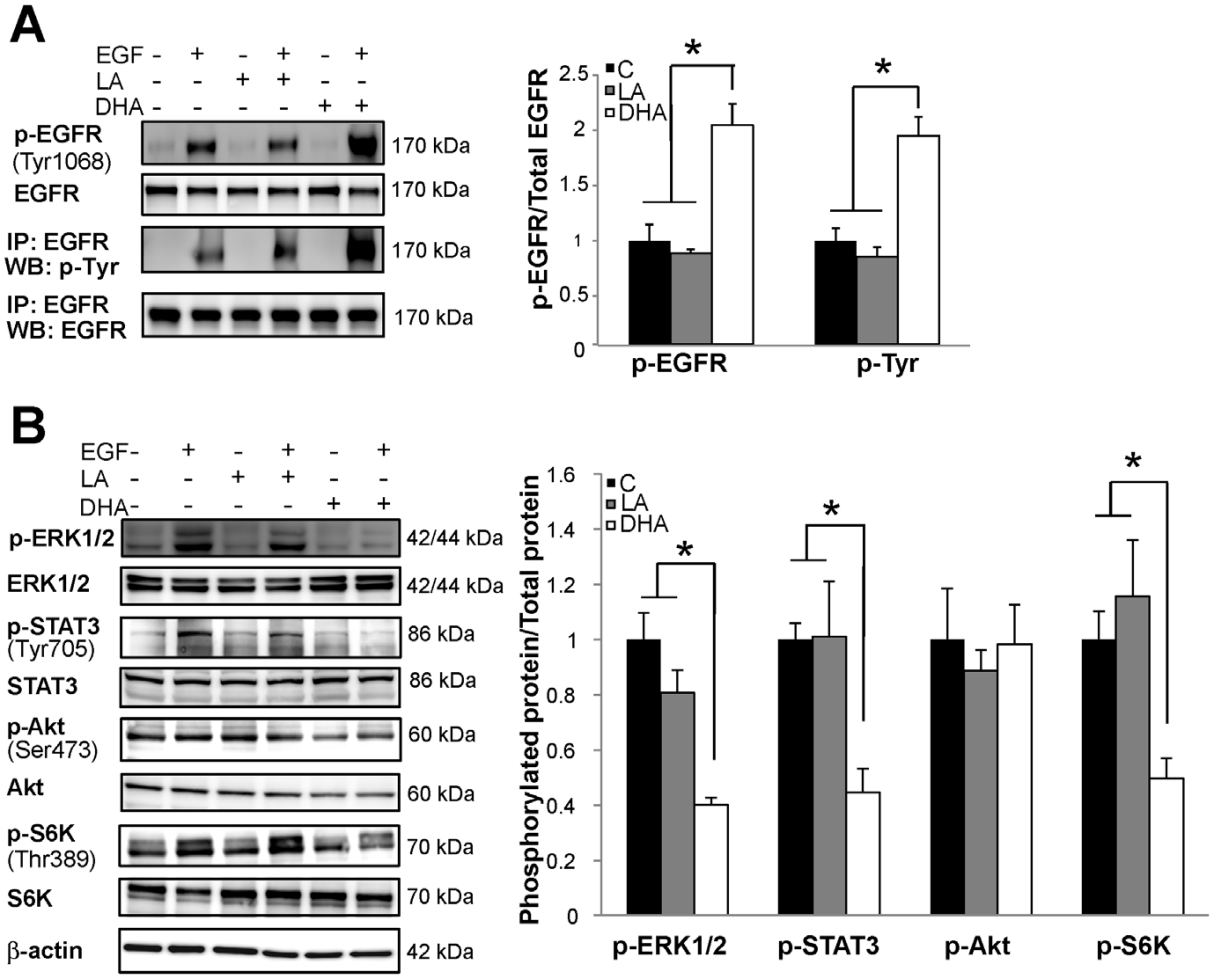
DHA increases EGFR phosphorylation but suppresses activation of downstream signaling. YAMC cells were untreated (control) or treated with 50 µM BSA-complexed fatty acids (LA or DHA) for 72 h. For the final 16–18 h, cells were incubated in low serum media (0.5% FBS) with the same concentration of fatty acids. Cells were either unstimulated or stimulated for 10 min with 25 ng/mL EGF and subsequently harvested. A) Equal amounts of protein from whole cell lysates were Western blotted for total and phosphorylated (Tyr1068) EGFR. Additionally, EGFR was immunoprecipitated from total cellular lysates prior to Western blotting for EGFR and phosphorylated tyrosine resides. B) Whole cell lysates were analyzed by Western blotting for total and phosphorylated downstream mediators of EGFR signaling, including ERK1/2, STAT3, S6kinase, and Akt. Each blot is representative of 3–4 independent experiments with 3 replicates of each treatment per experiment. Quantification of band volume was performed and data are presented as mean±SEM. Data are expressed as the ratio of the phosphorylated protein to total protein and normalized to control. Statistical significance between treatments (*P<0.05; **P<0.01) was determined using ANOVA and Tukey’s test of contrast. C, control; LA, linoleic acid; DHA, docosahexaenoic acid.

Due to the role that lipid rafts play in the regulation of EGFR activation and downstream signaling, we next investigated the effects of DHA on EGFR activation status and signaling in YAMC cells. Untreated and fatty acid treated cells were incubated in low serum media (0.5% FBS) overnight in order to reduce signaling from growth factors within the serum. Subsequently, cells were stimulated with 25 ng/mL EGF for 10 min followed by isolation of cell lysates. To assess the effects of fatty acid treatment on EGF-induced EGFR phosphorylation, lysates were probed for EGFR phosphorylated on Tyr1068, one of the major sites of EGFR phosphorylation that is involved in activation of downstream signaling. DHA treatment resulted in a greater than two-fold increase in EGFR phosphorylation compared to untreated control or LA treated cells (Figure 2A). EGFR was also immunoprecipitated from cell lysates followed by immunoblotting for total phosphorylated tyrosine residues. Consistent with the initial results, DHA enhanced total EGFR tyrosine phosphorylation by greater than two-fold (Figure 2A). Since EGFR phosphorylation is characteristically associated with activation of downstream signaling, we further probed cell lysates for phosphorylation of downstream effector proteins, including ERK1/2, STAT3, and Akt. DHA treatment resulted in a ∼50% reduction in the activation of ERK1/2 and STAT3 in DHA treated cells compared to untreated control and LA treated cells (Figure 2B). DHA had no effect on Akt phosphorylation at Ser473 (Figure 2B). The mammalian target of rapamycin (mTOR) is a serine/threonine kinase that is often hyperactivated in cancer, and stimulation of both Akt and ERK1/2 can lead to activation of a protein complex containing mTOR, i.e., mTORC1. To assess mTORC1 activity, we measured phosphorylation of ribosomal S6 kinase (S6K), a kinase that is directly phosphorylated by mTOR at Thr389. We found that DHA significantly suppressed EGF-induced phosphorylation of S6K, indicating reduced activation of mTOR (Figure 1C). Together, these data suggest that DHA disrupts the EGFR signaling cascade in colonocytes by disengaging EGFR phosphorylation from signal transduction.

**Figure 3:**
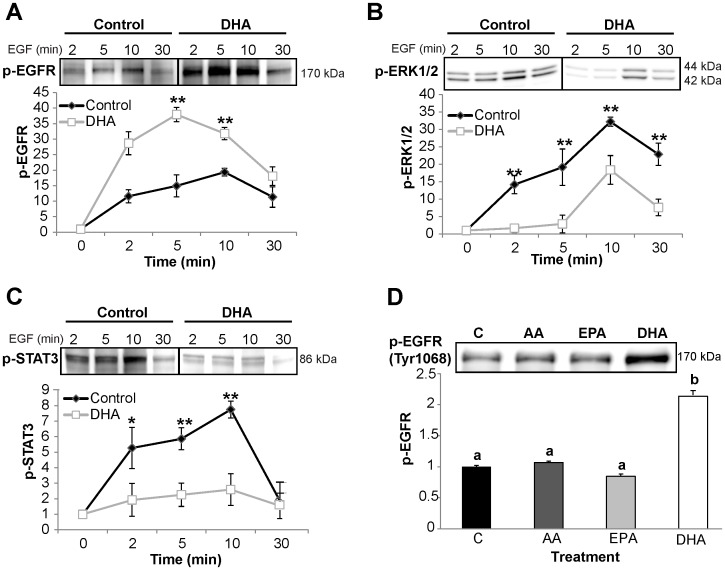
DHA uniquely alters EGFR function. YAMC cells were treated with control or 50 µM BSA-complexed fatty acids (LA or DHA) for 72 h. For the final 16–18 h, cells were incubated in low serum media (0.5% FBS) with the same concentration of fatty acids. Cells were stimulated for 0–30 min with 25 ng/mL EGF and subsequently harvested. Equal concentrations of protein from the whole cell lysates were analyzed by Western blotting for A) phosphorylated EGFR, B) phosphorylated ERK1/2, and C) phosphorylated STAT3. Each immunoblot is representative of 3 independent experiments. Quantification of band volume was performed and data are presented as mean±SEM and normalized to time 0 (n = 3). Statistical significance between treatments (*P<0.05; **P<0.01) was determined using Student’s *t*-test. D) Additionally, YAMC cells were treated with 50 µM BSA-complexed fatty acids (AA, EPA, or DHA) for a total of 72 h. For the final 16–18 h, cells were incubated in low serum (0.5% FBS) then stimulated for 10 min with 25 ng/mL EGF. Whole cell lysates were separated assessed by Western blotting for phosphorylated (Tyr1068) EGFR. A representative blot from 3 independent experiments is presented. Data are expressed as mean±SEM of phosphorylated EGFR, normalized to control (n = 3). Statistical significance between treatments (*P*<0.05) is indicated by different letters and was determined using ANOVA and Tukey’s test of contrast. C, control; LA, linoleic acid; DHA, docosahexaenoic acid; AA, arachidonic acid; EPA, eicosapentaenoic acid.

We also investigated the effect of DHA on EGFR phosphorylation and signaling at multiple time points. Untreated control and DHA treated cells were stimulated with 25 ng/mL EGF from 0–30 min. Consistent with initial results, EGFR phosphorylation was significantly increased by DHA treatment at 2, 5, and 10 min following stimulation (Figure 3A). EGFR phosphorylation peaked in DHA treated cells at 5 min and started decreasing, whereas EGFR phosphorylation was highest at 10 min in control samples, suggesting a dynamic alteration in EGFR regulation. Downstream signaling from EGFR through ERK1/2 and STAT3 was suppressed by DHA at each time point assessed (Figure 3B,C).

### Effect of Fatty Acid on EGFR is DHA Specific

Since DHA and EPA are both long chain omega-3 fatty acids that have been shown to reduce colon tumor development, we examined whether each of these fatty acids cause an increase in EGFR phosphorylation or if the effect is specific to DHA. We also assessed the effects of arachidonic acid (AA; 20∶4 n−6) on EGFR activation as a control, long-chain n-6 PUFA. Lysates were probed for phosphorylated EGFR. Neither AA nor EPA treatment resulted in an increase in EGFR phosphorylation compared to control (Figure 3D). Only DHA treatment increased the phosphorylation status of EGFR.

### Mechanism of DHA-induced Increase in EGFR Phosphorylation

**Figure 4:**
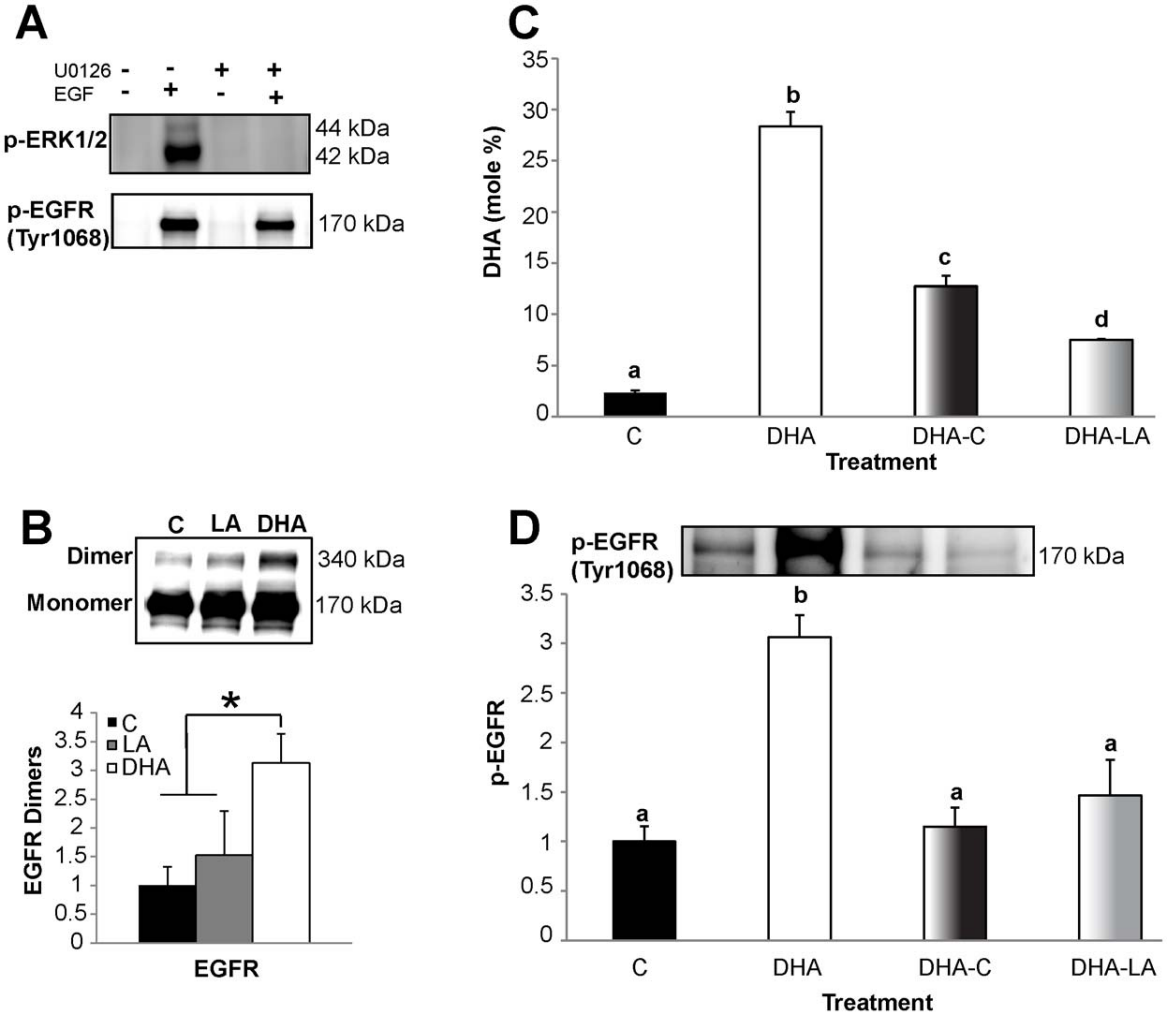
DHA alterations to EGFR function require enrichment of DHA in the plasma membrane. A) Control treated YAMC cells were incubated with low serum media overnight. Select cultures were then treated with 10 µM U0126 for 2 h. Untreated and U0126 treated cultures then underwent stimulation with 25 ng/mL EGF for 10 min. Lysates were assessed by Western blotting for phosphorylation of Erk1/2 and EGFR (Tyr 1068). B) Following stimulation with EGF, cells were subjected to chemical crosslinking prior to harvesting cell lysates. Cell lysate was assessed by Western blotting for EGFR. EGFR dimers were identified as bands with twice the molecular weight of EGFR monomers. C-D) For “wash out” experiments, YAMC cells were either untreated or treated with 50 µM DHA for 72 h. Select DHA treated cultures were then washed and incubated for an additional 40 h with either untreated media or 50 µM LA. For the final 16–18 h, cells were incubated with low serum media (0.5% FBS) followed by stimulation with 25 ng/mL EGF then harvested. C) Membrane lipids were extracted and enrichment of membrane phospholipids with DHA was quantified by capillary gas chromatography/mass spectrometry. D) Lysates were assessed by Western blotting for phosphorylated EGFR (Tyr1068). The blot is representative of 3 independent experiments. Data are presented as mean±SEM of phosphorylated EGFR, normalized to control. Statistical significance between treatments as indicated by different letters (*P*<0.05) was determined using ANOVA and Tukey’s test of contrast. C, control; AA, arachidonic acid; EPA, eicosapentaenoic acid; DHA, docosahexaenoic acid; LA, linoleic acid.

EGFR phosphorylation is controlled by a variety of factors. Therefore, we next determined the mechanism by which DHA increases EGFR phosphorylation. Previous studies have suggested a number of possible contributing mechanisms. For example, EGFR phosphorylation has been shown to be increased by an adaptive mechanism in response to suppression of downstream signaling through ERK1/2 [61]. Another study demonstrated that the lipid environment directly influences EGFR dimerization, which results in increased EGFR phosphorylation [62]. Since we demonstrated that DHA both alters the membrane environment of EGFR and suppresses ERK1/2 phosphorylation, we next assessed whether either of these two mechanisms contributed to the observed DHA-induced increase in EGFR phosphorylation. First, we utilized a specific inhibitor of ERK1/2, U0126, to recapitulate the suppressive effect of DHA on ERK1/2 activation. We then assessed the effect of this inhibitor on EGFR activation status. We found that treatment of cells with 10 µM U0126 for 2 h prior to stimulation completely inhibited EGF-induced activation of ERK1/2 (Figure 4A). However, this inhibition had no effect on overall EGFR phosphorylation, suggesting that a downstream feedback-mediated increase in EGFR phosphorylation induced by ERK1/2 suppression is not a mechanism by which DHA enhances EGFR phosphorylation.

We subsequently measured the effect of DHA on EGFR dimerization. Following stimulation with EGF, EGFR dimers were linked using bis[sulfosuccinimidyl] suberate (BS^3^), a non-permeable crosslinking reagent. DHA treated cells exhibited a greater than three-fold increase in dimerization compared to control and LA treated cells (Figure 4B). This is consistent with previous studies indicating that the lipid environment affects EGFR dimerization [62], suggesting that the effect of DHA on EGFR is due to the alteration in the lipid environment of the receptor, resulting in increased EGFR dimerization and, therefore, phosphorylation.

We postulated that if the DHA-induced adaptive phosphorylation of EGFR is a result of membrane enrichment causing disruption of lipid rafts, then the effect should be reversible by depleting the cells of DHA. To test this hypothesis, cells were treated with DHA, followed by a washout period wherein the cells were either treated with no fatty acid or with LA. As expected, the washout period resulted in significantly reduced levels of DHA in the plasma membrane (Figure 4C), and ligand-induced EGFR phosphorylation was normalized back to the same level as control (Figure 4D). These data indicate that the inhibition of EGFR signaling by DHA is dependent on its presence in the plasma membrane.

### DHA Inhibits EGF-stimulated Ras GTP-binding

**Figure 5:**
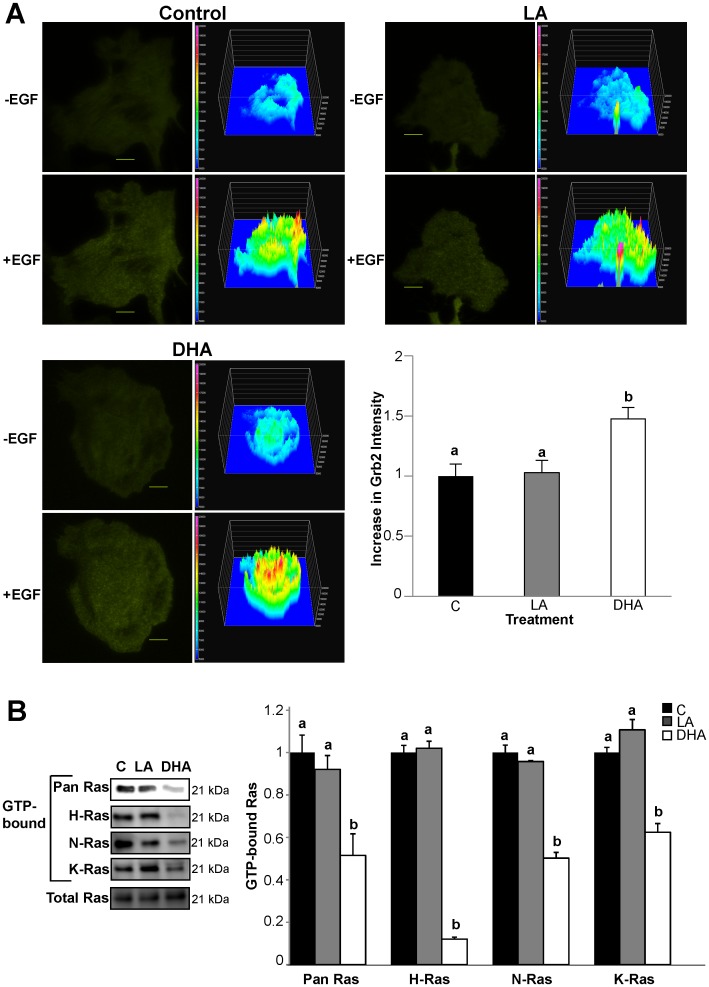
DHA impairs EGF-induced activation of Ras. A) YAMC cells were treated with 50 µM BSA-complexed fatty acids for 72 h. Twenty-four h after initiating fatty acid treatment, cells were transfected with Grb2-YFP. For the final 16–18 h, cells were incubated with low serum media (0.5% FBS) with the same concentration of fatty acids and imaged using TIRF microscopy. Cells were stimulated with 100 ng/mL EGF and imaged every 5 sec. Images are representative of 4 independent experiments (n = 22−25 cells/treatment). Changes in total surface intensity were quantified using Nikon Elements AR 3.2. Fluorescence images and the respective surface intensity plots are shown. Surface intensity plots were generated in Nikon Elements AR 3.2 (the scale is from blue (lowest intensity) to red/pink (highest intensity). Data are presented as mean±SEM normalized to control. Bars, 10 µM. B) YAMC cells were treated with 50 µM BSA-complexed fatty acids for 72 h. For the final 16–18 h, cells were incubated with low serum media (0.5% FBS) with the same concentration of fatty acids. Cells were stimulated with 25 ng/mL EGF for 2 min and harvested. GTP-bound Ras was isolated using a GST pull-down assay. Isolated GTP-bound Ras was then analyzed by Western blotting for pan Ras. Isolated GTP-bound Ras was additionally analyzed by Western blotting for H, K, and N-Ras. Blots are representative of 3 independent experiments. Quantification of band volume was performed. Data are expressed as mean±SEM (n = 3), normalized to control. Statistical significance between treatments (*P*<0.05) as indicated by different letters was determined using ANOVA and Tukey’s test of contrast. C, control; LA, linoleic acid; DHA, docosahexaenoic acid.

Due to the fact that DHA treatment increased EGFR phosphorylation while concurrently suppressing activation of downstream mediators, we attempted to pinpoint the site of perturbation in the signaling cascade. Since the EGFR-Ras-ERK1/2 pathway is well documented, we examined the components of this pathway downstream of the receptor. Following EGF stimulation, growth receptor-bound protein 2 (Grb2) is recruited to the plasma membrane and binds to phosphorylated tyrosine residues of EGFR (Tyr1068 and Tyr1086). To assess recruitment of this immediate proximal signal downstream of EGFR phosphorylation, cells were transfected with fluorescently-tagged SH2 domain of Grb2 (Grb2-YFP), capable of binding to the phosphorylated tyrosine residues of EGFR. Cells were subsequently imaged using total internal reflective fluorescence (TIRF) microscopy to assess Grb2-YFP translocation to the plasma membrane in response to EGFR activation. Following stimulation with EGF, Grb2-YFP was rapidly recruited to the plasma membrane (Figure 5A). We observed a significant increase in EGF-stimulated Grb2-YFP plasma membrane translocation in DHA treated cells compared to control and LA treated cells. Grb2 recruits Son of Sevenless (Sos), a guanine nucleotide exchange factor (GEF), to activate Ras. Therefore, we assessed Ras activation status by pulling-down GTP-bound Ras followed by Western blotting for Ras. DHA-treated cells had significantly lower levels of GTP-bound Ras compared to control and LA-treated cells (Figure 5B). This observation indicates that the DHA-induced perturbation in the EGFR-Ras-ERK1/2 pathway occurs at the site of Ras activation.

Ras is comprised of three distinct isoforms, including H-Ras, K-Ras, and N-Ras. Isoform-specific signaling is regulated by differential compartmentalization within cell surface microdomains and intracellular compartments [60]. H-Ras is enriched in lipid rafts, both caveolar and noncaveolar, whereas K-Ras is exclusively located in the bulk membrane [59], [60]. Additionally, N-Ras is mainly segregated into noncaveolar lipid rafts [63]. Furthermore, Ras proteins have been shown to signal from the Golgi complex, the endoplasmic reticulum, and endomembranes, e.g., endosomes (9, 26, 32). The effect of this distinctive segregation on the signals generated by Ras is just beginning to unfold. Therefore, we assessed the effect of DHA on activation of each Ras isoform to provide further clarity into the mechanism by which DHA suppressed EGF-induced Ras activation. We found that DHA suppressed activation of all three isoforms of Ras, indicating a common mechanism by which DHA suppresses EGF-induced activation of Ras. Furthermore, activation of H-Ras was almost entirely inhibited by DHA, suggesting a distinct mechanism of regulation for this specific isoform.

### DHA Induces Increased EGFR Internalization and Degradation

**Figure 6:**
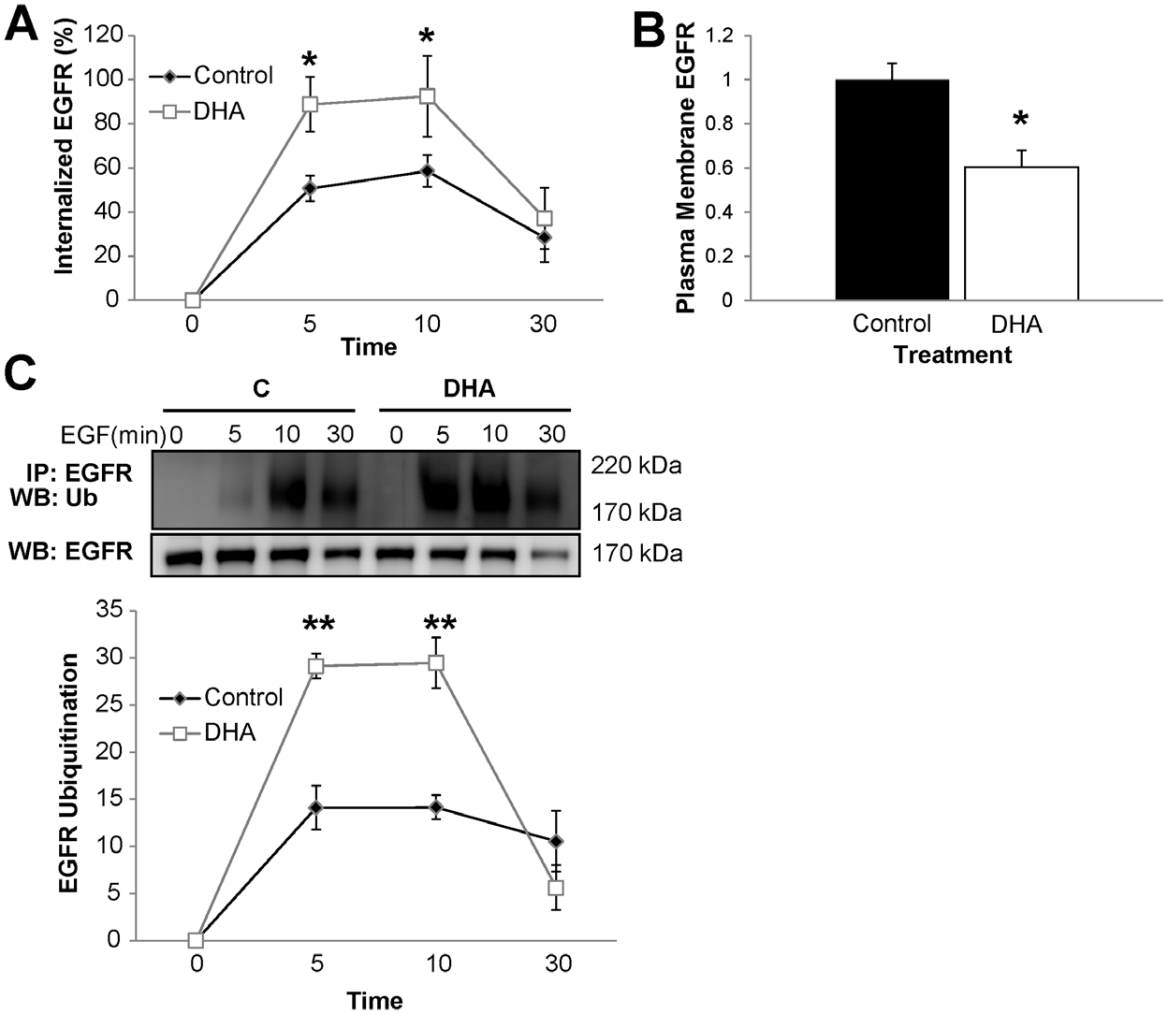
DHA mediates increased EGFR internalization and degradation. YAMC cells were incubated with untreated media or media supplemented with 50 µM BSA-complexed DHA for a total of 72 h. For the final 16–18 h, cells were incubated with low serum media (0.5% FBS). A) Cell surface proteins were labeled with EZ-Link Sulfo-NHS-SS-Biotin followed by stimulation with 25 ng/mL EGF for 0–30 min. After stimulation, cells were washed and biotin remaining on the cell surface was cleaved. Cell lysates were harvested, and biotinylated EGFR was quantified by ELISA using streptavidin coated plates and anti-EGFR antibody. B) Cell surface EGFR was assessed by treating cells the same as in A, and harvesting without stimulating with EGF or cleaving cell surface biotin. C) Cells were stimulated with 25 ng/mL EGF for 0–30 min and harvested. EGFR was immunoprecipitated from the total cell lysate, assessed by Western blotting for ubiquitin, and quantification of band intensity was performed. All results are representative of at least 3 independent experiments. Data represent mean±SEM. In (A) and (C), data are normalized to time 0. In B), data are normalized to control (no fatty acid). Statistical significance between treatments (*P<0.05. **P<0.01) was determined using Student’s *t*-test. C, control; DHA, docosahexaenoic acid.

Endocytosis is a major mechanism of EGFR signal attenuation by targeting the activated receptor for lysosomal proteolysis [64]. Recent evidence suggests that lipid rafts may play a role in mediating EGFR endocytosis [65], [66]. Additionally, EGFR phosphorylation and Grb2 recruitment to EGFR are intimately linked to EGFR internalization [67], [68], [69], [70]. Therefore, we next assessed the effect of DHA on EGFR endocytosis using a surface biotinylation assay. Interestingly, internalization of EGFR occurred more rapidly in DHA treated cells compared to untreated (control) cells (Figure 6A). In addition, consistent with data in Figure 4, A and B, DHA reduced the steady-state plasma membrane localization of EGFR in unstimulated cells (Figure 6B). Following internalization, EGFR can either be recycled back to the cell surface or be targeted to the lysosome for degradation. Ubiquitin acts as a sorting signal to down-regulate the functions of plasma membrane proteins. EGFR internalization and degradation are facilitated by ubiquitin [71]. Receptor endocytosis stimulated by ubiquitination is considered to be crucial to prevent oncogenesis because it sorts the receptor to the lumen of multivesicular bodies and terminates growth factor signaling [72]. This is especially crucial for EGFR since it has been shown to continue to signal from endosomes after it is internalized [67]. Therefore, we assessed ubiquitination of the receptor in order to determine whether the DHA-induced increase in EGFR internalization was associated with increased receptor ubiquitination and degradation. Immunoprecipitation of EGFR followed by Western blotting for ubiquitin to assess EGFR ubiquitination was utilized. We found that EGFR ubiquitination was significantly increased by DHA treatment (Figure 6C). Additionally, EGFR expression was reduced in DHA treated cells 30 min after stimulation. Together, these data suggest that DHA further regulates EGFR signaling capacity by increasing EGFR internalization and degradation.

### Suppression of Cell Proliferation by DHA is EGFR-dependent

**Figure 7:**
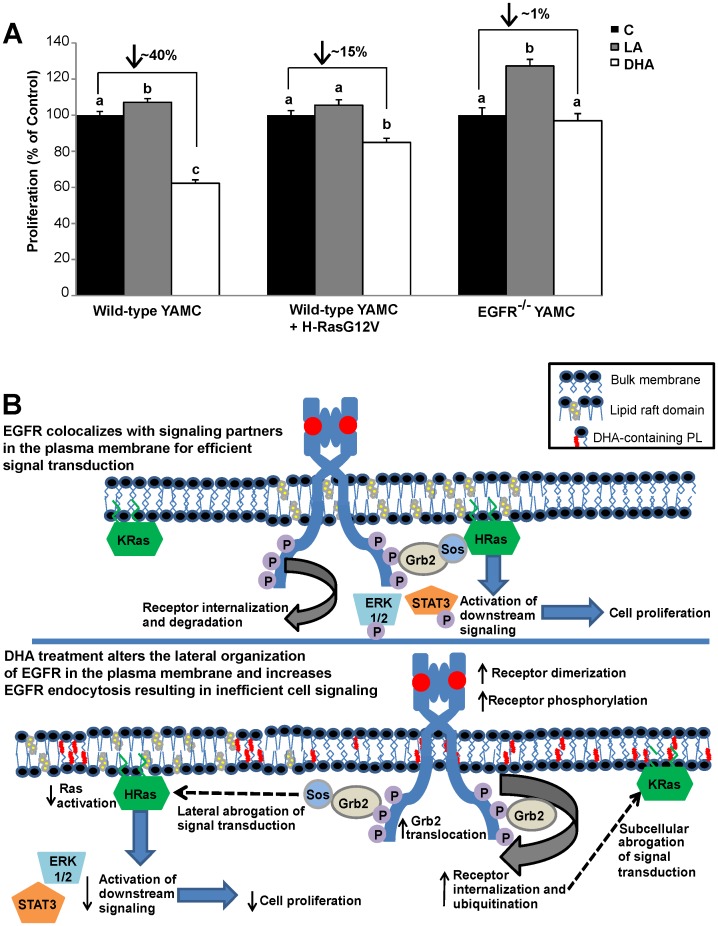
DHA suppresses EGFR-mediated cell proliferation. A) Wild-type and EGFR^−/−^ YAMC cells were treated with 50 µM BSA-complexed fatty acid for 24 h. Following the initial treatment, cells were seeded at an equal density into a 96-well plate, and cultured under the same treatment conditions for an additional 48 h. Select cultures were transfected using nucleofection with GFP-H-RasG12V prior to being seeded into the 96-well plate. Cell proliferation was measured using CyQUANT cell proliferation assay. Results are representative of 3 independent experiments. Data are expressed as mean±SEM (n = 9 samples per treatment). Statistical significance between treatments as indicated by different letters (*P*<0.05) was determined using ANOVA and Tukey’s test of contrast. No comparisons were made between proliferation of wild-type and EGFR^−/−^ cells. B) Putative model: Under control conditions, EGFR is enriched in liquid ordered lipid raft domains of the cell. Upon stimulation, ligand bound receptors dimerize and transphosphorylate. The phosphorylated tyrosine residues then serve as docking sites for adaptor proteins, which leads to the activation of downstream targets, including Ras. Signaling is efficient due to the lateral organization of EGFR and downstream signaling partners. Upon fatty acid treatment, the cell incorporates DHA into plasma membrane phospholipids. DHA is sterically incompatible with cholesterol and perturbs liquid ordered domains resulting in a change in the lateral organization of EGFR. Ligand-induced receptor dimerization and phosphorylation is increased, along with receptor ubiquitination, internalization, and degradation. The altered lateral and subcellular organization of EGFR results in inefficient cell signaling. Suppression of EGFR signaling causes altered cellular function, including reduced cell proliferation.

In order to document a functional endpoint of the DHA-induced decrease in EGFR signaling, we chose to measure cell proliferation, which is regulated in part by signaling through EGFR. DHA treatment of wild-type YAMC cells resulted in an approximately 40% decrease in cell proliferation compared to control and LA treated cells (Figure 7A). We additionally assessed whether expression of a constitutively active form of H-Ras (GFP-H-RasG12V) could rescue the DHA-induced suppression of cell proliferation. We found that DHA-treated cells expressing constitutively activated H-Ras recovered partially from the DHA-induced suppression of cell proliferation but still exhibited approximately a 15% decrease in cell proliferation compared to control cells expressing GFP-H-RasG12V. Additionally, the effect of DHA on proliferation of an isogenic cell line that does not express EGFR (EGFR^−/−^) was assessed. Interestingly, DHA treatment had no effect on cell proliferation in EGFR^−/−^ YAMC cells compared to control. Together, these data demonstrate that DHA suppresses cell proliferation in an EGFR-dependent manner. Based on these findings, we have developed a putative model illustrating the effects of DHA on EGFR (Figure 7B).

### Fish Oil Inhibits EGFR Signaling in Mouse Colonic Mucosa

**Figure 8:**
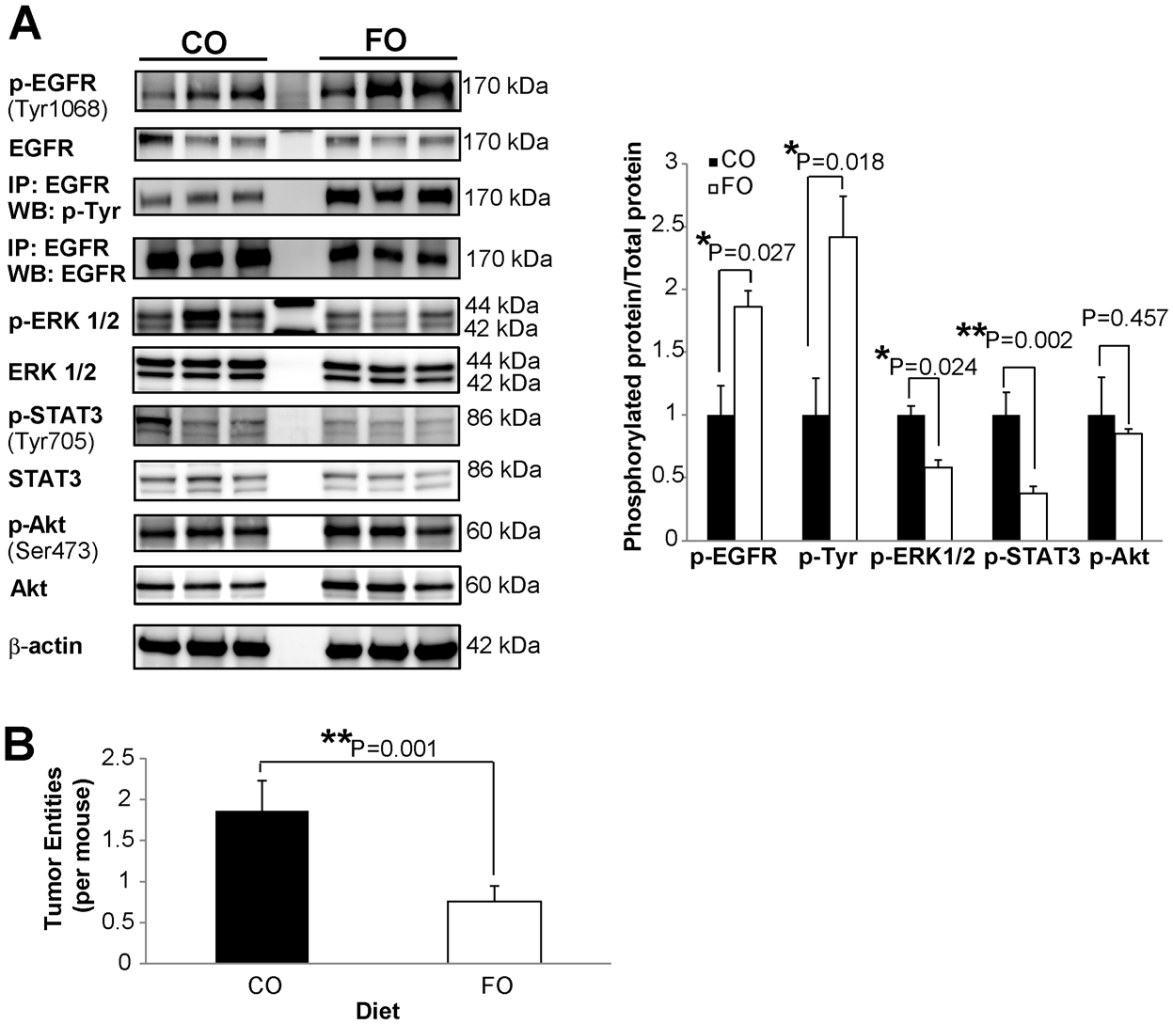
Fish oil feeding suppresses colonic epithelial EGFR signaling in mice. Carcinogen (AOM) and DSS-treated mice were fed a diet enriched in fish oil (high in DHA) or corn oil (contains no DHA) for a total of 15 weeks. A) Whole cell lysates were isolated from scraped colonic mucosa. Equal concentrations of protein were assessed by Western blotting for total and phosphorylated EGFR (Tyr1068). Additionally, EGFR was immunoprecipitated from mucosal lysates and Western blotted for phosphorylated tyrosine residues and EGFR. Also, whole cell lysates were probed by Western blotting for total and phosphorylated downstream mediators of EGFR signaling, including ERK1/2, STAT3, and Akt. Quantification of band volume was performed and data are presented as mean±SEM of the ratio of phosphorylated protein to total protein and normalized to CO, n = 12 mice per diet. B) Colon lesions were fixed in 4% paraformaldehyde, embedded in paraffin, stained with hematoxylin-eosin, and evaluated by a board certified pathologist. The mean colon tumor entity number, including adenomas and adenocarcinomas, per mouse in each diet treatment is presented, n = 22−25 mice per diet. Statistical significance between diets was determined using Student’s *t*-test. CO, corn oil; FO, fish oil.

In order to further corroborate the effects of DHA on EGFR activation status, we evaluated the status of total and phosphorylated EGFR in colonic mucosa from carcinogen-injected (azoxymethane), chronically-inflamed (dextran sulfate sodium treated) C57BL/6 mice fed diets enriched in fish oil (containing DHA) or corn oil (control, contains primarily LA and no n-3 polyunsaturated fatty acids). Protein lysates from the uninvolved colonic mucosa were collected and probed for total and phosphorylated EGFR (Tyr1068) by Western blotting. Additionally, we performed immunoprecipitation for EGFR followed by Western blotting for phosphorylated tyrosine residues. Consistent with the cell culture data, phosphorylation of EGFR increased approximately two-fold in mice fed a fish oil diet compared to mice fed a corn oil diet, while no difference in the total expression of EGFR was observed (Figure 8A). We further probed tissue lysates for phosphorylation of ERK1/2, STAT3, and Akt. Similar to Figure 1 data, phosphorylation of ERK1/2 and STAT3 decreased by approximately 40% and 50%, respectively, in fish oil fed mice compared to corn oil fed mice with no difference in phosphorylation of Akt between diets (Figure 8A). Additionally, tumors from the animals were stained and typed. Interestingly, fish oil fed animals developed fewer tumors compared to animals fed a corn oil diet (Figure 8B). Overall, these data indicate a protective effect of dietary fish oil against colon tumorigenesis by suppressing EGFR signal transduction.

## Discussion

EGFR is a transmembrane receptor tyrosine kinase involved in transmitting external cues to the intestinal epithelium, thereby modulating cell proliferation, migration, and survival. Recent evidence suggests that the membrane lipid microenvironment can significantly modulate EGFR localization and function [62]. Here, we report that membrane incorporation of DHA alters the lateral organization of EGFR (Figure 1). The lateral organization of the receptor has been shown to directly regulate ligand binding, dimerization, and receptor phosphorylation [5], [6], [9]. Consistent with these observations, we demonstrated that DHA treatment resulted in increased ligand-stimulated EGFR dimerization and phosphorylation (Figures 2A and 4B). Therefore, the DHA-induced shift of EGFR localization within the plasma membrane alters the ability of the receptor to dimerize and transphosphorylate. These findings favor a model according to which receptor modulation by fatty acids is mediated by the membrane.

According to the canonical process of signal transduction, the observed increase in EGFR phosphorylation upon treatment with DHA is expected to be correlated with enhanced downstream signaling. Two previous studies have observed that DHA increases EGFR phosphorylation, but they reported conflicting results regarding downstream signaling [54], [55]. Additionally, the flawed design of one of the studies renders the results difficult to interpret. Therefore, in our study, we evaluated the effect of DHA on activation of multiple distinct signaling cascades that radiate from EGFR within the colon. Although DHA was found to increase EGFR phosphorylation, we noted that this fatty acid uniquely inhibited EGF-stimulated activation of downstream signaling from EGFR through ERK1/2, STAT3, and mTOR/S6K (Figure 2B). A substantial body of work has documented the central role of lateral membrane organization in mediating EGFR signal transduction [73]. Integral to EGFR signaling is its localization to lipid raft domains. These specialized membrane domains have the ability to assemble the molecular machineries necessary for intracellular propagation of EGFR effector signals. Stimulation with EGF induces coalescence of lipid raft domains and promotes the formation of these signaling platforms, which suggests a central role for these domains in EGFR signal propagation [74]. Collectively, these data suggest that by altering the lateral organization of EGFR, DHA can modulate multiple cellular signaling cascades.

In addition to regulating the localization of EGFR within the plasma membrane, DHA also altered the subcellular distribution of EGFR. Under unstimulated conditions, DHA treated cells contained significantly less EGFR at the plasma membrane (Figure 6B). Additionally, concomitant with increased EGFR phosphorylation, DHA treated cells exhibited enhanced EGFR endocytosis following receptor stimulation (Figure 6A). Because a majority of signaling cascades are activated by EGFR at the plasma membrane, receptor endocytosis and degradation play important roles in terminating EGFR signaling. Therefore, the DHA-mediated enhancement of EGFR internalization is an additional mechanism by which DHA can suppress the ability of EGFR to activate downstream signaling. Once internalized, EGFR can continue signaling from endosomes, recycle back to the plasma membrane, or traffic for degradation. This is noteworthy because DHA increased EGFR ubiquitination and degradation (Figure 6C). Interestingly, although DHA treatment did not affect the steady-state levels of EGFR, following ligand-induced activation, it significantly increased EGFR ubiquitinatation and degradation. Since receptor endocytosis and degradation can function to mitigate signal transduction, we propose that this is an additional mode by which DHA modulates the signaling capacity of EGFR.

To further understand the mechanism by which DHA suppresses EGFR signal transduction, we attempted to identify the exact locus of the DHA-induced perturbation in EGFR signal transduction. Therefore, we assessed the individual components of the EGFR-Ras-ERK1/2 signaling axis. Grb2 is an immediate proximal downstream mediator of EGFR signaling and directly binds to phosphorylated tyrosine residues of EGFR, including Tyr1068, which we showed here to be increased by DHA treatment. Grb2 mediates multiple responses to EGFR phosphorylation, including propagation of intracellular signaling and receptor endocytosis. We observed that DHA treatment increased the EGF-stimulated recruitment of Grb2 to the plasma membrane (Figure 5A), indicating that DHA does not affect the ability of phosphorylated EGFR to recruit intracellular signaling mediators. We subsequently demonstrated that the breakpoint in the DHA-mediated suppression of EGFR signaling was at the level of Ras activation (Figure 5B). Our lab has previously shown that DHA suppresses the levels of activated GTP-bound Ras under basal conditions [75]. We have extended these observations by demonstrating that EGFR-mediated activation of all three isoforms of Ras was suppressed by DHA. In addition, DHA had the strongest inhibitory effect on activation of H-Ras. This is intriguing due to the fact that both H-Ras and EGFR are localized to lipid rafts [59], and that EGF-stimulated interactions between Ras and its downstream partner Raf-1 have been shown to occur in lipid rafts [3]. A potential explanation for the observed DHA-mediated suppression of activation of Ras, specifically H-Ras, is based on the altered localization of EGFR within the plasma membrane. Convincing evidence illustrates the importance of lateral segregation of signaling cascades on the plasma membrane. Indeed, for signal transduction to occur, phosphorylated EGFR must form a transient complex with Grb2, Sos, and Ras [76]. Due to the altered plasma membrane localization of EGFR upon treatment with DHA, this could result in a spatial abrogation of signal transduction by reducing the formation of these transient complexes.

Since DHA antagonizes the activation of all Ras isoforms regardless of their plasma membrane localization, it is likely that an additional mechanism mediates the suppression of EGFR signal transduction by DHA. Therefore, we hypothesize that the observed increased endocytosis of the receptor contributes to the suppressive phenotype. The majority of activation of Ras by EGFR has been shown to occur at the plasma membrane [77]. Therefore, the observed DHA-induced increase in EGFR internalization likely contributes to the reduced activation of all Ras isoforms. We are currently determining whether the altered plasma membrane localization of EGFR and/or enhanced EGFR endocytosis are responsible for the suppression of downstream signaling. Additionally, further work is required to determine how the altered plasma membrane localization of EGFR contributes to enhanced receptor internalization and degradation. Previous work by Sigismund et al. indicates that high levels of EGFR phosphorylation can lead to activation of additional forms of EGFR endocytosis which sort EGFR for degradation instead of recycling [66]. Therefore, it is possible that DHA could function by altering EGFR plasma membrane localization, resulting in higher levels of EGFR phosphorylation and activation of a non-canonical form of receptor endocytosis that sorts the receptor for degradation as opposed to recycling. Current studies in our lab are focused on testing this hypothesis.

These results have substantial biological relevance because Ras plays a central role in the development of human colon cancer and is commonly hyperactivated by somatic mutation or signaling through growth factor receptors [78]. Hence, by suppressing EGF-stimulated activation of Ras, DHA can provide protection against colonic transformation. In addition to suppressing activation of the Ras-ERK1/2 pathway, DHA suppressed EGF-stimulated activation of STAT3. STAT3 can be activated downstream of EGFR by several pathways, and Ras signaling has been shown to be intimately linked to STAT3 activation [79], [80], [81]. Therefore, the reduction in STAT3 activation could be a direct result of reduced activation of Ras. Additionally, lipid rafts have been shown to play a central role in the activation of STAT3 [82], which may explain the DHA-induced suppression of STAT3 activation. Clearly, further studies are required to determine the exact mechanism of action.

To analyze the consequence of the suppression of EGFR signaling by DHA, we measured cell proliferation in wild-type and EGFR-null colonocytes. Interestingly, DHA suppressed cell proliferation only in cells with a functional EGFR (Figure 7A). These results indicate that the DHA modulates cell proliferation in an EGFR-dependent manner. It is important to note that DHA does not induce apoptosis in this cell line [83], so the changes observed are due to suppressed proliferation and not increased cell death. Additionally, expression of a constitutively active form of H-Ras partially rescued the DHA-induced suppression of cell proliferation. The limited ability of constitutively active H-Ras was likely due to the inhibitory effect that DHA has on other downstream pathways from EGFR that are independent of Ras signaling. Based on these data, we have developed a putative model according to which DHA alters the cellular localization and signaling capacity of EGFR (Figure 7B). By altering the lateral organization of EGFR, DHA enhances the ability of EGFR to dimerize and transphosphorylate. DHA also increases EGFR internalization and degradation, which suppresses the ability of EGFR to activate downstream signaling cascades.

In order to determine the *in vivo* effect of DHA on EGFR signaling, we assessed whether dietary consumption of fish oil, enriched in DHA, modulates EGFR phosphorylation and signaling in mice. Consistent with our cell culture data, feeding a DHA-enriched diet resulted in an increase in EGFR phosphorylation and a suppression of ERK1/2 and STAT3 activation in mouse colonic epithelium (Figure 8). The suppression of EGFR signaling in the colon was associated with a reduction in tumor-incidence in mice fed a DHA-enriched diet. These data highlight the unique mechanism by which DHA suppresses colon tumorigenesis.

The differential effects of DHA and EPA, the two n-3 PUFAs enriched in fish oil, are often overlooked and underappreciated. Therefore, we assessed whether EPA had the same effect as DHA on EGFR phosphorylation. Interestingly, in contrast to DHA, neither EPA nor AA, another long-chain PUFA, exerted an effect on EGFR phosphorylation (Figure 3D). This is consistent with a previous study showing that DHA, but not EPA, suppressed EGF-stimulated activation of AP-1 [84]. Collectively, these results highlight the uniqueness of DHA, which has been shown to significantly alter numerous membrane properties [48], [49]. The effects of DHA on EGFR signaling are reversible when supplementation with DHA is discontinued and the fatty acid is washed out of the plasma membrane (Figure 4D). This is consistent with our hypothesis that DHA enrichment in the plasma membrane directly modulates EGFR signaling. DHA is a structurally unique fatty acid. It is slightly polar due to its six double bonds, and it rapidly reorients through multiple conformational states [48]. This flexible structure renders DHA incompatible with ordered saturated acyl chains and cholesterol, two major constituents of lipid rafts. Multiple biophysical studies utilizing model membranes and molecular dynamic simulations have shown that DHA acyl chains do not pack efficiently with cholesterol and saturated acyl chains [85], [86]. Additionally, studies using cell lines have demonstrated that DHA can disrupt lipid rafts and alter protein distribution [52], [53], [87]. Although, the exact effect that DHA has on lipid rafts remains to be fully elucidated, an abundance of data clearly demonstrate that DHA can impede lipid raft mediated processes and alter lipid raft composition [47], [54]. Our data demonstrate that DHA inhibits the lateral organization of EGFR within the plasma membrane. Furthermore, the data suggest that DHA modifies lipid rafts because it altered the overall localization of the lipid raft marker, RFP-tH, within the plasma membrane. Overall, this work provides further evidence of the global effect of DHA on lipid rafts.

A seminal finding of this work is the DHA-induced severance of EGFR phosphorylation from downstream signaling. Phosphorylation of EGFR has previously been considered a marker for activation of downstream signaling. In contrast, our data describe a mechanism whereby DHA induces an increase in EGFR phosphorylation by altering its localization but suppresses downstream signaling. Further understanding of the relationship between plasma membrane composition and receptor organization is required to fully elucidate the regulation of cell signaling. The interactions between cellular membranes and signaling proteins are emerging as important for mediation of cellular signaling [88], [89]. Indeed, cell surface spatio-temporal organization is fundamentally dependent on the targeting of proteins to specialized membrane domains, as well as the dynamics of these protein-membrane associations. Ultimately, the intimate relationship of signaling proteins and membranes enables intricate control and refinement of biological processes. Based on the work presented here, fatty acids, specifically DHA, play a central role in regulating signal transduction by altering membrane interaction dynamics.

## Materials and Methods

### Cell Culture

Young adult mouse colonic (YAMC) cells, conditionally immortalized colonocytes, were originally obtained from R.H. Whitehead, Ludwig Cancer Institute (Melbourne, Australia). Both wild-type and EGFR^−/−^ isotype YAMC cells were utilized. YAMC cells (passages 12–17) were cultured under permissive conditions, 33°C and 5% CO_2_ in RPMI 1640 media (Mediatech, Manassas, VA) supplemented with 5% fetal bovine serum (FBS; Hyclone, Logan, UT), 2 mM GlutaMax (Gibco, Grand Island, NY), 5 µg/mL insulin, 5 µg/ml transferrin, 5 ng/ml selenious acid (Collaborative Biomedical Products, Bedford, MA), and 5 IU/mL of murine interferon-γ (Roche, Mannheim, Germany). Select cultures were treated for 72 h with 50 µM fatty acid [DHA, linoleic acid (LA, 18∶2n−6), arachidonic acid (AA, 20∶4n−6), or eicosapentaenoic acid (EPA, 20∶5n−3); NuChek, Elysian, MN] complexed with bovine serum albumin (BSA). Where indicated, DHA treated cultures were washed three times with PBS followed by incubation with untreated media or LA for an additional 40 h. In select cultures, for the final 16–18 h, complete media was replaced with low-serum (0.5% FBS) media. Cells were then stimulated with 0–25 ng/mL recombinant mouse EGF (Sigma, St. Louis, MO) and harvested. In select cultures, cells were incubated with the ERK1/2 inhibitor U0126 (Invitrogen, Grand Island, NY) at 10 µM for 2 h prior to stimulation with EGF.

### Lipid Raft Isolation

To determine the localization of EGFR within the plasma membrane, YAMC cells were treated with fatty acid and incubated with low serum media as above. Detergent-free lipid raft-enriched fractions were isolated as previously described [57], [58]. All steps were performed at 4°C. YAMC cells grown in 12 T-175 flasks per treatment were harvested with trypsin-EDTA (Gibco) and pelleted at 200×*g* for 5 min. The pellets were resuspended in Buffer A (250 mM sucrose, 1 mM EDTA, 20 mM tricine, 100 M activated sodium orthovanadate, 40 L/mL protease cocktail, pH 7.8) at 1×10^7^ cell/mL. Cells from each treatment were pooled and lysed by two rounds of rapid freeze (−80°C) and thaw (37°C). Cell lysates were then centrifuged at 1000×*g* for 10 min, and the supernatants were retained. Cell pellets were resuspended in Buffer A and homogenized as above, and the centrifugation step was repeated. The resulting supernatants were pooled into a post-nuclear supernatant (PNS). The PNS was layered on top of 30% Percoll (Amersham, Pittsburg, PA) in Buffer A and centrifuged at 84,000×*g* for 30 min in a Beckman SW28 rotor. The plasma membrane fraction was collected and sonicated 3 times (50-J bursts) with 2 rapid pulses each time. The samples were then mixed with OptiPrep (Accurate Chemical and Scientific Corp, Westbury, NY) in Buffer A (final concentration 23%), overlaid with a 6 mL linear 20 to 10% OptiPrep gradient, and centrifuged in a Beckman SW41Ti rotor at 52,000×*g* for 90 min. The top 5 mL of the gradient was collected and combined with 4 mL of 50% OptiPrep in Buffer A. An aliquot of the denser membrane band (HDM) was collected. The 9 mL fraction was overlaid with 1 mL of 15% Optiprep in Buffer A and 0.5 mL of 5% Optiprep in Buffer A and was centrifuged at 52,000 *g* for 90 min in a Beckman SW41Ti rotor. A lipid raft/caveolae-enriched membrane fraction (LR) was collected from the 5/15% interface, and a membrane fraction defined as the intermediate fraction (IDM) was collected at the bottom of the 15% layer. Slide-a-lyser cassettes (Pierce, Rockford, IL) were used to dialyze samples overnight in dialysis buffer (1 mM EDTA, 20 mM tricine, pH 7.8). Samples were placed into 1.5 mL Eppendorf tubes and centrifuged in a SpeedVac System to 1/3 the original volume to concentrate. Protein concentration was measured with Coomassie Plus Protein assay (Pierce), and fractions were subjected to SDS-PAGE and Western blotting as described above.

### Colocalization

The plasmid containing RFP conjugated with a truncated H-Ras composed of the C-terminal 9 amino acid targeting domain (RFP-tH) was a generous gift from Ian Prior, Univ. of Liverpool [90]. The plasmid containing full length human EGFR conjugated to GFP containing a point mutation of A206K in the GFP sequence to prevent GFP dimerization (EGFR-mGFP) was a kind gift from Hung-Jun Liao, Vanderbilt University [91]. Cells were seeded at a density of 1.0×10^5^ cells per well into Lab-Tek II 2-well chambered coverglass slides (Nalge Nunc, Rochester, NY) 24 h prior to transfection and cultured in complete RPMI media containing 50 µM fatty acid. Cells were cotransfected with 0.3 µg RFP-tH and 1.5 µg EGFR-mGFP using Effectene (Qiagen, Valencia, CA) in media without fatty acid according to the manufacturer’s instructions. Transfection conditions were optimized to minimize the amount of DNA and lipofection reagent used to avoid nonspecific cytotoxicity. Four h after transfection, cells were washed and the media was replaced and supplemented with 50 µM fatty acid for 48 h. Cells were incubated in low serum media (0.5% FBS) with fatty acid for the final 16–18 h prior to imaging. Cells were imaged 48 h after transfection. Prior to imaging, cultures were washed with Leibovitz’s L-15 medium (Gibco) followed by addition of 1 mL of Leibovitz’s media (without serum) per well. Images were collected with a Zeiss 510 META NLO Multiphoton Microscope System consisting of an Axiovert 200 MOT microscope (Carl Zeiss Microimaging, Thornwood, NY) equipped with an argon laser, PMT, and LSM software. For EGFR-mGFP and RFP-tH, excitation wavelengths of 488 nm and 543 nm were used, and fluorescence emission was monitored at 530 nm and 590 nm, respectively. Images were collected in confocal mode with the pinhole set at 1 AU using a 40× objective (1.3 NA oil immersion lens) at room temperature. Identical acquisition parameters were used for all images within the experiment. Colocalization at the plasma membrane was analyzed by quantifying Mander’s colocalization coefficient for green (EGFR-mGFP) with red (RFP-tH) using Nikon Elements AR 3.2. Analysis was performed on background-subtracted 16-bit images.

### Western Blotting

For Western blotting, cells were homogenized in ice-cold homogenization buffer (50 mM Tris-HCl, pH 7.2, 250 mM sucrose, 2 mM EDTA, 1 mM EGTA, 50 mM sodium fluoride, 100 mM sodium orthovanadate, 1%Triton X-100, 100 µM activated sodium orthovanadate, 10 mM β-mercaptoethanol, and protease inhibitor cocktail) as previously described [92]. Following homogenization, lysates were sheared using a 29G needle, incubated on ice for 30 min, and centrifuged at 16,000×*g* for 20 min. The supernatant was collected and protein concentration was assessed using Coomassie Plus Protein assay (Pierce). Lysates were treated with 1× pyronin sample buffer and subjected to SDS polyacrylamide gel electrophoresis (PAGE) in precast 4–20% Tris-glycine mini gels (Invitrogen). After electrophoresis, proteins were electroblotted onto a polyvinylidene fluoride membrane with the use of a Hoefer Mighty Small Transphor unit at 400 mA for 90 min. Following transfer, the membrane was incubated in 5% BSA (Roche) and 0.1% Tween 20 in TBS (TBST) at room temperature for 1 h with shaking, followed by incubation with shaking overnight at 4°C with primary antibody diluted in 5% BSA in TBST. Membranes were washed with TBST and incubated with secondary peroxidase conjugated secondary antibody as per manufacturer’s instructions. Bands were developed using Pierce SuperSignal West Femto^TM^ maximum sensitivity substrate. Blots were scanned using a Fluor-S Max MultiImager system (Bio-Rad, Hercules, CA). Quantification of bands was performed using Quantity One software (Bio-Rad). Monoclonal rabbit anti-EGFR, phospho-EGFR (Tyr1068), Akt, phospho-Akt (Ser473), ERK1/2, p-ERK1/2, STAT3, phospho-STAT3 (Tyr705), p70 S6kinase, and phospho-p70 S6kinase (Thr389) and monoclonal mouse phospho-tyrosine were purchased from Cell Signaling (Danvers, MA). Monoclonal mouse anti-ubiquitin and polyclonal rabbit anti-K, H, and N-Ras were purchased from Santa Cruz (Santa Cruz, CA). Affinity purified mouse anti-clathrin heavy chain and purified mouse anti-caveolin 1 were purchased from BD Transduction Laboratories (Bedford, MA). Peroxidase conjugated goat anti-rabbit IgG was purchased from Kirkegaard and Perry Laboratories (Gaithersburg, MD), and peroxidase conjugated goat anti-mouse IgG was purchased from Jackson ImmunoResearch (West Grove, PA). For detection of ganglioside GM-1, peroxidase conjugated cholera toxin B subunit was purchased from Sigma.

### Immunoprecipitation

Cell lysates were incubated with rotation purified polyclonal rabbit anti-EGFR antibody (Millipore, Billerica, MA) overnight. The protein-antibody conjugates were then pulled down using protein-G conjugated Dynabeads (Invitrogen). Protein was eluted from the dynabeads using 2× pyronin buffer, and equal volumes of the samples were run on SDS-PAGE and Western blotted for EGFR, phospho-tyrosine, or ubiquitin. Lysates analyzed for ubiquitination of EGFR were treated with 5 mM N-ethylmaleimide to prevent post lysis deubiquitination of EGFR [61].

### Receptor Dimerization

To assess EGFR dimerization, cells were treated as above. Following low serum incubation, cells were washed with ice-cold PBS prior to incubation with 0 or 25 ng/mL EGF on ice for 1 h. Cells were then washed with ice-cold PBS followed by incubation on ice for 20 min with 3 mM bis(sulfosuccinimidyl) suberate (BS^3^, Pierce), a non-permeable crosslinking reagent. In all experiments, a freshly prepared solution of BS^3^ was used. The crosslinking reaction was quenched by adding 250 mM glycine in PBS and further incubation on ice for 5 min. Cells were washed with PBS and homogenized as above. Protein concentration was measured and lysates were assessed by Western blotting for EGFR as described above.

### Fatty Acid Analysis

To assess incorporation of DHA into phospholipids, membrane lipids were extracted with chloroform-methanol (2∶1 vol/vol), and total phospholipids were separated by thin-layer chromatography with chloroform-methanol-acetic acid-water (90∶8:1∶0.8 vol/vol/vol/vol). After transesterification, fatty acid methyl esters were quantified by capillary gas chromatography/mass spectrometry [93].

### Total Internal Reflective Fluorescence (TIRF) Microscopy

A plasmid containing the SH2 domain of Grb2 conjugated to YFP (Grb2-YFP) was generously provided by Alexander Sorkin, University of Colorado [94]. For TIRF experiments, cells were treated with fatty acids for 24 h prior to transfection. Cells were transfected using Amaxa nucleofection kit L (Amaxa, Basel, Switzerland) with 2.0 µg Grb2-YFP. Following transfection, cells were seeded at a density of 25,000 cells/well into MatTek (Ashland, MA) glass bottom 35 mm dishes in the presence of fatty acid. Approximately 32 h after transfection, cells were incubated in low serum media (0.5% FBS) containing fatty acid for 16–18 h prior to imaging. Images were acquired on a Zeiss TIRF Microscope system consisting of a Zeiss AxioObserver Z1 microscope (Carl Zeiss) equipped with a high resolution AxioCam MRm camera, argon laser, and AxioVision 4 software. An excitation wavelength of 514 nm was used, and emission was monitored at 537 nm. All images were collected using a 100× objective (1.4 NA oil immersion) at 37°C and 5% CO_2_. To observe the translocation of Grb2-YFP to the plasma membrane, cells were stimulated with 100 ng/mL EGF, and images were collected every 5 sec. Images were collected using identical image acquisition parameters for all images within the experiment. Whole cell fluorescence intensity was performed on background-subtracted 16-bit images using Nikon Elements AR 3.2.

### Ras Activation Assay

To assess the activation status of Ras, YAMC cells were treated for 72 h with fatty acid and incubated with low serum media (0.5% FBS) for the final 16–18 h. Cells were stimulated with 25 ng/mL EGF for 2 min, then harvested using Pierce cell lysis buffer. Activated (GTP-bound) Ras was subsequently isolated and assessed by Western blotting using the Pierce Active Ras Pull-Down and Detection^TM^ kit according to the manufacturer’s instructions. Additionally, Ras isoforms were detected by Western blotting the isolated GTP-bound Ras using isoform specific antibodies as described above.

### Biotinylation

To assess cell surface EGFR and receptor internalization, cells were treated with fatty acids, incubated with low serum media and surface biotinylated using thiol-cleavable EZ-Link Sulfo-NHS-SS-Biotin (Pierce), 0.5 mg/mL, dissolved in PBS for 30 min on ice. Labeled cells were then rinsed two times with ice-cold PBS, and excess biotin was quenched with 60 mM iodoacetamide in PBS buffer for 5 min at 4°C. Select cultures were harvested at this step in order to quantify cell surface expression of EGFR. Cells were then washed three times with ice-cold PBS, followed by incubation at 33°C in prewarmed serum-free RPMI media for 5 min followed by stimulation with 25 ng/mL EGF for 0–30 min. Biotin groups remaining on the cell surface were then cleaved off by three 20 min washes with buffer containing reducing agent [100 mM MESNA (sodium-2-mercaptoethane sulfonate), 50 mM Tris (pH 8.6), 100 mM NaCl, 1 mM EDTA, and 0.2% BSA] at 4°C. Cells were then washed three times in ice-cold PBS and lysed in RIPA buffer (20 mM Tris, 150 mM NaCl, 2 mM EDTA, 2 mM EGTA, 1% sodium deoxycholate, 1% SDS, and 1% Triton X-100) containing protease and phosphatase inhibitors. The lysates were clarified by centrifugation at 16,000×*g* for 20 min. Protein concentration was assessed using BCA protein assay (Pierce). Biotinylated EGFR was captured on streptavidin ELISA plates (NUNC IMMOBILIZER Streptavidin C8) from the cell lysates diluted to 5 µg/mL total protein in PBS containing 0.5% Tween 20, pH 7.3 (PBST), during a 2 h incubation at room temperature on a shaker. Plates were then washed three times with PBST, incubated with anti-EGFR antibody (Santa Cruz) (2 µg/mL) for 2 h at room temperature, washed, and incubated with horseradish peroxidase-conjugated secondary antibody for 1 h at room temperature. The plates were subsequently washed three times in PBST before adding color substrate (R&D Systems, Minneapolis, MN) for 5–10 min. Color development was stopped by addition of an equal amount of 4 M H_2_SO_4_ and analyzed at 450 nm.

### Cell Proliferation

YAMC cells (both wild-type and EGFR^−/−^) were treated with fatty acids for 24 h prior to being seeded into a 96-well plate at a density of 1.0×10^5^ cells per well. Cells were cultured in complete media supplemented with fatty acids for an additional 48 h. Additionally, wild-type YAMC cells were transfected using nucleofection (Amaxa kit L) with a constitutively active form of H-Ras (H-RasG12V-GTP), provided by Dr. Ian Prior [95], prior to being seeded into a 96 well plate and cultured for 48 h. Cells were then washed with PBS and cell proliferation was measured using CyQuant cell proliferation assay (Molecular Probes, Grand Island, NY) according to the manufacturer’s instructions.

### Mice

Female C57BL/6 wild-type mice were used. All procedures followed guidelines approved by the U.S. Public Health Service and the Institutional Animal Care and Use Committee at Texas A&M University. This study and all animal protocols and procedures were approved by the Institutional Animal Care and Use Committee at Texas A&M University (Permit number: AUP2010-079). All efforts were made to minimize suffering. Mice were maintained under barrier conditions and initially consumed a Teklad commercial mouse non-purified diet (Harlan, Indianapolis, IN) ad libitum. Mice were maintained for 15 weeks on a semi-purified defined diet that was adequate in all nutrients, containing 320 (g/kg diet) sucrose, 200 casein, 220 corn starch, 3 DL-methionine, 35 AIN 76 salt mix, 10 AIN 76 mineral mix, 2 choline chloride, 60 cellulose (Bio-Serv, Frenchtown, NJ), 150 corn oil (CO) (Dyets, Bethlehem, PA) or 115 vacuum-deodorized Menhaden fish oil (FO) (Omega Protein, Houston, TX) plus 35 CO. The animals were provided the defined diet 2 weeks prior to an injection of azoxymethane (AOM; 7.5 mg/kg body weight). One week after the AOM injection, mice were exposed to 3 cycles of 1% dextran sulfate sodium (DSS) for 4 d followed by 17 d of recovery. Mice were sacrificed 6 weeks after the last DSS treatment (13 weeks post AOM injection) by CO_2_ asphyxiation. Colon lesions were mapped and excised prior to colonic mucosa isolation by scraping, and the mucosa was snap frozen in liquid nitrogen and stored at -80°C until homogenization. Lesions were fixed in 4% paraformaldehyde, embedded in paraffin, stained with hematoxylin-eosin, and evaluated in a blinded manner by a board-certified pathologist. For immunoblotting, mucosa was homogenized in ice-cold homogenization buffer as described above. For immunoprecipitation, mucosa was homogenized using Pierce Classic^TM^ IP lysis buffer according to the manufacturer’s instructions. Following homogenization, lysates were sheared using a 29G needle, incubated on ice for 30 min, and then centrifuged at 16,000×*g* for 20 min. The supernatant was collected. Protein concentration was determined using Coomassie Protein Plus assay (Pierce).

### Statistics

The effect of 2 independent variables (treatment effects) was assessed using Student’s t-test. The effect of more than 2 independent variables (treatment effects) was assessed using the one-way analysis of variance test (ANOVA), and differences among means were evaluated using Tukey’s post-hoc test of contrast. *P* values <0.05 were considered to be statistically significant.
